# Accurate and Practical Energy Detection over α-μ Fading Channels

**DOI:** 10.3390/s20030754

**Published:** 2020-01-29

**Authors:** Kaitian Cao, Ping Qian, Jing An, Li Wang

**Affiliations:** 1 School of Electrical & Electronic Engineering, Shanghai Institute of Technology, Shanghai 201418, China; 2 Key Laboratory of Broadband Wireless Communication and Sensor Network Technology, Ministry of Education, Nanjing University of Posts and Telecommunications, Nanjing 210003, China

**Keywords:** wireless sensor network, cognitive radio network, energy detection, Meijer’s G-function, α-μ fading channels

## Abstract

In this study, a novel and exact closed-form expression for detection probability of energy detection (ED) in terms of Meijer’s G-function over α-μ generalized fading channels was derived. It is more accurate and practical than the existing exact expressions and has wide application prospects in the performance evaluations in various areas of wireless communications, especially in the wireless sensor network (WSN) and the cognitive radio network (CRN). Furthermore, an exact and simple analytical solution for the sample size meeting the desired detection performance in terms of the probability mass function of a Poisson distribution was also solved. Simulations verified the detection performance and accuracy of our derived expressions with a small sample size compared to the existing exact expressions and approximations.

## 1. Introduction

Currently, much attention is being attached to the energy detection (ED) algorithm by researchers [1,2], and ED is still a promising technique for signal detection in the wireless sensor network (WSN) and cognitive radio network (CRN) since it does not require prior information about PU (primary user)’s signals and has low implementation complexity. Detection performance of ED is mainly evaluated by false alarm probability ($P_{f}$) and detection probability ($P_{d}$). In fading channel, the detection performance of ED is fundamentally measured by the average detection probability (${\bar{P}}_{d}$) owing to the fluctuant signal-to-noise ratio (SNR).

As CRs must detect potential spectrum opportunities rapidly, ED detectors must operate with the fewest possible samples while offering high detection reliability [2]. In addition, $P_{d}$ is an intractable and complex generalized Marcum Q-function, and it is very difficult to find simple and tractable closed-form expressions for $P_{d}$ or ${\bar{P}}_{d}$. Therefore, many ED approximations based on small sample size such as Central limit theorem (CLT) [1] and Sankaran’s third approximation (STA) [2] have been presented for rapid detection. Nevertheless, all the approximations are only aimed at the performance analyses of ED over the AWGN channel, not over the generalized fading channels. Furthermore, ED approximations cannot obtain the exact closed-form expression for the sampling number that satisfies the detection performance.

The α-μ distribution is a general and flexible fading distribution for channel models, which includes some important distributions such as Gamma, Nakagami-m, exponential, Weibull, one-sided Gaussian, and Rayleigh [3]. Recently, some exact closed-form expressions for ${\bar{P}}_{d}$ over α-μ generalized fading channels were investigated [3,4,5]. However, all these expressions are complex and impractical since they all include infinite series and require truncation in practice. Furthermore, all these exact ED methods still do not investigate the simple and exact analytical expressions for the number of samples that achieves the required detection performance due to infinite series. To deal with these problems, in this paper we aimed to study a novel and exact closed-form expression for ${\bar{P}}_{d}$ over α-μ generalized fading channels. The main contributions of this paper are summarized as follows:

(i) Unlike the existing exact expressions of ${\bar{P}}_{d}$ containing infinite series that are intractable and impractical, an exact and practical closed-form expression (without infinite series) for ${\bar{P}}_{d}$ over α-μ fading channels is proposed.
(ii) An exact and simple analytical solution for the sample size achieving the desirable detection performance over α-μ fading channels was also obtained. To the best of our knowledge, the exact and simple expression for the sample size has never been addressed in the existing literature.
(iii) The performance of our ED method under small sample size was verified through Monte-Carlo simulations compared to the existing exact and approximate ED methods.

## 2. Conventional Energy Detection (CED)

In this paper, we assume that the fading channel coefficients remain constant for all samples. Therefore, the signal detection at SU (secondary user) can be modeled as a binary hypothesis testing problem as follows:

(1) $$\left\{ \begin{array}{ll} x(n)=\eta (n) & H_{0}\\ x(n)=s(n)+\eta (n) & H_{1} \end{array}\right.$$

where x(n) is the observation from SU at instant *n* (*n* = 1, 2 …, *N*), η(n) is the AWGN noise with mean zero and variance $\sigma_{\eta }^{2}$. s(n) is the PU signal sampled by SU at instant *n*. For brevity, s(n) is assumed as a random signal with mean zero and variance $\sigma_{s}^{2}$. Suppose that s(n) is independent of η(n). The hypotheses $H_{0}$ and $H_{1}$ represent the absence and the presence of the PU, respectively.

In CED, the normalized test statistic T(x) can be represented as $T(x)=\sum_{n=1}^{N}{\left|x(n)/\sigma_{\eta }\right|}^{2}$, where *N* is the number of samples. Therefore, the false alarm probability ($P_{f}$) and the detection probability ($P_{d}$) can be represented as follows:

(2) $$P_{f}=P\left(T(x)\geq \lambda |H_{0}\right)=\frac{\Gamma (N,\lambda /2)}{\Gamma (N)}$$

(3) $$P_{d}=P\left(T(x)\geq \lambda |H_{1}\right)=Q_{N}(\sqrt{2\gamma },\sqrt{\lambda })$$

where $\gamma =\sigma_{s}^{2}/\sigma_{\eta }^{2}$ denotes the SNR, $\Gamma (a,b)=\int_{b}^{\infty }t^{a-1}e^{-t}dt$ and $\Gamma (a)=\int_{0}^{\infty }t^{a-1}e^{-t}dt$ represent the upper incomplete Gamma function and the Gamma function, respectively. $Q_{n}(a,b)=\int_{b}^{\infty }\frac{x^{n}}{a^{n-1}}e^{-(x^{2}+a^{2})/2}I_{n-1}(ax)dx$ is the *n*-th order generalized Marcum Q-function with the *m-*th order modified Bessel function of the first kind: $I_{m}(y)=\sum_{k=0}^{\infty }\frac{{\left(y/2\right)}^{2k+m}}{k!\Gamma (k+m+1)}$ [6], where a>0 and b≥0. Note that $P_{f}$ is independent of γ.

Under hypothesis *H*_0_, 2T(x) follows the central chi-squared distribution with 2*N* degrees of freedom [7], and hence the expression of $P_{f}$ can be derived as

(4) $$P_{f}=1-F_{\chi_{2N}^{2}}\left(\lambda \right)$$

where $F_{\chi_{2N}^{2}}(y)=\left(\int_{0}^{\frac{y}{2}}t^{N-1}e^{-t}dt\right)/\Gamma (N)$ is the regularized lower incomplete gamma function. Thus,

(5) $$\lambda =F_{\chi_{2N}^{2}}^{-1}(1-P_{f})$$

where $F_{\chi_{2N}^{2}}^{-1}(\cdot )$ is the inverse of $F_{\chi_{2N}^{2}}(\cdot )$.

Similarly, under hypothesis *H*_1_, 2T(x)/(1+γ) follows the non-central chi-squared distribution with 2*N* degrees of freedom, thus the expression of $P_{d}$ can be derived as

(6) $$P_{d}=\frac{\Gamma \left[N,\lambda /\left(2(1+\gamma )\right)\right]}{\Gamma (N)}=1-F_{\chi_{2N}^{2}}\left(\frac{\lambda }{1+\gamma }\right)$$

Thus, from (4) and (6), the Receiver Operating Characteristic (ROC) can be easily derived as

(7) $$P_{d}=1-F_{\chi_{2N}^{2}}\left({\left(1+\gamma \right)}^{-1}F_{\chi_{2N}^{2}}^{-1}(1-P_{f})\right)$$

Notably, it is very hard to obtain a tractable and accurate closed-form expression of $P_{d}$ directly from (3) due to the complexity and intractability of the generalized Marcum-Q function. In addition, an exact closed-form expression of sample size (*N*) in terms of $P_{d}$ and $P_{f}$ can hardly be derived since (7) is impossible to be converted with respect to *N*.

## 3. The Proposed Analytic Closed-Form Solutions for ${\bar{P}}_{d}$ and Minimum Sample Size

The probability density function (PDF) of the instantaneous SNR γ in *α*-*μ* fading channels [8] with envelope *R* is given as

(8) $$f_{\alpha -\mu }(\gamma )=\frac{\alpha \gamma^{\alpha \mu /2-1}\mu^{\mu }}{2{\bar{\gamma }}^{\alpha \mu /2}\Gamma (\mu )}\exp\left(-\mu \frac{\gamma^{\alpha /2}}{{\bar{\gamma }}^{\alpha /2}}\right)$$

where $\alpha ,\mu \in {\mathbb{Z}}^{+}$ and $\bar{\gamma }=\sqrt[{\alpha }]{E(R^{\alpha })}$(E(⋅) is the expectation operator) represent the non-linearity of the medium, the number of multipath clusters and average SNR, respectively.

**Proposition** **1.** *For $\bar{\gamma },\lambda \in {\mathbb{R}}^{+}$ and $\alpha ,\mu ,N\in {\mathbb{Z}}^{+}$, the following closed-form expression for the average detection probability of ED over α-μ fading channels is valid*

(9a) $$f_{\alpha -\mu }(\gamma )=\frac{\alpha \gamma^{\alpha \mu /2-1}\mu^{\mu }}{2{\bar{\gamma }}^{\alpha \mu /2}\Gamma (\mu )}\exp\left(-\mu \frac{\gamma^{\alpha /2}}{{\bar{\gamma }}^{\alpha /2}}\right)$$

(9b) $$n_{\lambda ,N}=\frac{e^{-\lambda /2}(\lambda /2{)}^{N}}{N!}$$

(9c) $$c_{\alpha ,\mu ,\bar{\gamma }}=\sum_{m=0}^{\mu -1}\frac{1}{m!}\frac{\sqrt{2\alpha }\mu^{m}}{{\left(2\pi \right)}^{\alpha /2}}{\left(\frac{\alpha }{\bar{\gamma }}\right)}^{\frac{\alpha m}{2}}G_{\begin{array}{cc} \alpha & 2 \end{array}}^{\begin{array}{cc} 2 & \alpha \end{array}}\left[\frac{\mu^{2}}{4}{\left(\frac{\alpha }{\bar{\gamma }}\right)}^{\alpha }|\begin{array}{c} \Delta \left(\alpha ,-\alpha m/2\right)\\ \Delta (2,0) \end{array}\right]$$

*where $G_{\begin{array}{cc} \varepsilon & \zeta \end{array}}^{\begin{array}{cc} i & j \end{array}}\left[\cdot \right]$ is Meijer’s G-function [9], $\Delta (s,t)=\frac{t}{s}$, $\frac{t+1}{s}$, …, $\frac{t+s-1}{s}$ ([10], Equation (22)). Note that $c_{\alpha ,\mu ,\bar{\gamma }}$ is only related to α, μ and $\bar{\gamma }$, whereas $n_{\lambda ,N}$ is independent of these parameters. Obviously, the above exact closed-form expressions for the average detection probability of ED does not contain any infinite series. Therefore, it is tractable and simple in practical scenarios.*

**Proof.**  From (8), we can obtain the average probability of detection over *α*-*μ* fading channels as

(10) $$\begin{array}{ll} {\bar{P}}_{d} & =\int_{0}^{\infty }P_{d}(\gamma )f_{\alpha -\mu }(\gamma )d\gamma =\int_{0}^{\infty }Q_{N}(\sqrt{2\gamma },\sqrt{\lambda })f_{\alpha -\mu }(\gamma )d\gamma \\ & =\int_{0}^{\infty }\frac{\alpha \mu^{\mu }}{2\Gamma (\mu )}\frac{\gamma^{\alpha \mu /2-1}}{{\bar{\gamma }}^{\alpha \mu /2}}Q_{N}(\sqrt{2\gamma },\sqrt{\lambda })\exp\left(-\mu \frac{\gamma^{\alpha /2}}{{\bar{\gamma }}^{\alpha /2}}\right)d\gamma \end{array}$$

Letting $\sqrt{\gamma /\bar{\gamma }}=x$, then (10) can be simplified to

(11) $${\bar{P}}_{d}=A\int_{0}^{\infty }x^{k-1}Q_{N}(cx,b)\exp(-px^{q})dx$$

where $A=\alpha \mu^{\mu }/\Gamma (\mu )$, k=αμ, $c=\sqrt{2\bar{\gamma }}$, $b=\sqrt{\lambda }$, p=μ, q=α. Letting $v=Q_{N}(cx,b)$, $du=x^{k-1}\exp(-px^{q})dx$, and applying integration by parts to (11), namely, ${\bar{P}}_{d}=A\left[{uv|}_{x=0}^{\infty }-\int_{0}^{\infty }udv\right]$. Thus, the following task is to find *dv* and *u*. With the help of ([6], Equation (4.33)) then evaluating *dv* as

(12) $$\begin{array}{ll} dv=dQ_{N}(cx,b) & =\int_{b}^{\infty }y^{N}e^{-\frac{y^{2}}{2}}d\left[e^{-\frac{{\left(cx\right)}^{2}}{2}}\underset{A_{1}}{\underset{︸}{{\left(cx\right)}^{-(N-1)}I_{N-1}(cxy)}}\right]dy\\ & =\int_{b}^{\infty }y^{N}e^{-\frac{y^{2}}{2}}\left[-e^{-\frac{{\left(cx\right)}^{2}}{2}}c^{2}xA_{1}+e^{-\frac{{\left(cx\right)}^{2}}{2}}\frac{dA_{1}}{dx}\right]dxdy \end{array}$$

Applying the modified Bessel function of the first kind of order *m*: $I_{m}(z)=\sum_{k=0}^{\infty }\frac{{\left(z/2\right)}^{2k+m}}{k!\Gamma (k+m+1)}$, then $dA_{1}/dx$ can be derived as

(13) $$\frac{dA_{1}}{dx}=cy{\left(cx\right)}^{-(N-1)}I_{N}(cxy)$$

Substituting (13) into (12), we can obtain

(14) $$dv=c{\left(cx\right)}^{1-N}e^{-\frac{{\left(cx\right)}^{2}}{2}}\int_{b}^{\infty }y^{N}e^{-\frac{y^{2}}{2}}\left[yI_{N}(cxy)-cxI_{N-1}(cxy)\right]dydx$$

Making use of ([6], Equation (4.43)), (14) can be deduced to

(15) $$dv=2^{-N}c^{2}xe^{-\frac{{\left(cx\right)}^{2}}{2}}\left[\underset{A_{2}}{\underset{︸}{\int_{b}^{\infty }\frac{y^{2N+1}e^{-\frac{y^{2}}{2}}}{\Gamma (N+1)}dy}}-\underset{A_{3}}{\underset{︸}{\int_{b}^{\infty }\frac{2y^{2N-1}e^{-\frac{y^{2}}{2}}}{\Gamma (N)}dy}}\right]dx$$

Letting $z=y^{2}/2$, then

(16) $$A_{2}=2^{N}\frac{\Gamma (N+1,b^{2}/2)}{\Gamma (N+1)}$$

In the similar way

(17) $$A_{3}=2^{N}\frac{\Gamma (N,b^{2}/2)}{\Gamma (N)}$$

Inserting (16) and (17) into (15), and applying ([6], Equations (4.44) and (4.46)), then *dv* can be finally derived as

(18) $$dv=\underset{A_{4}}{\underset{︸}{\left[\frac{e^{-b^{2}/2}(b^{2}/2{)}^{N}}{N!}\right]}}c^{2}xe^{-\frac{{\left(cx\right)}^{2}}{2}}dx$$

*u* can be obtained by the indefinite integral in ([9], Equation (2.33.10)), given as

(19) $$u=-\frac{\Gamma \left(k/q,px^{q}\right)}{kp}$$

Substituting (18) and (19) into ${\bar{P}}_{d}=A\left[{uv|}_{x=0}^{\infty }-\int_{0}^{\infty }udv\right]$, and noting that $Q_{m}(0,\beta )=\frac{\Gamma (m,\beta^{2}/2)}{\Gamma (m)}$([6], Equation (4.44)) and $Q_{m}(\infty ,\beta )=1$ that can be readily deduced according to ([6], Equation (4.36)), thus (11) can be expressed as below

(20) $${\bar{P}}_{d}=A\left[\frac{\Gamma \left(k/q\right)}{kp}\frac{\Gamma \left(N,b^{2}/2\right)}{\Gamma \left(N\right)}+A_{4}\underset{A_{5}}{\underset{︸}{\int_{0}^{\infty }\frac{\Gamma \left(k/q,px^{q}\right)}{kp}c^{2}xe^{-\frac{{\left(cx\right)}^{2}}{2}}dx}}\right]$$

Letting $y={\left(cx\right)}^{2}/2$, then *A*_5_ can be reduced to

(21) $$A_{5}=\int_{0}^{\infty }\frac{\Gamma (k/q,\overset{B}{\overset{︷}{2^{q/2}pc^{-q}}}y^{q/2})}{kp}e^{-y}dy$$

Noting that $\Gamma \left(v,z\right)=\Gamma \left(v\right)-\int_{0}^{z}t^{v-1}e^{-t}dt$, k/q=μ∈ℕ, and applying ([9], Equation (2.321.2)), ([10], Equation (21)) and ([11], Equation (8)), we have

(22) $$A_{5}=\frac{\Gamma \left(k/q\right)}{kp}\sum_{m=0}^{k/q-1}\frac{1}{m!}B^{m}\frac{\sqrt{2}q^{\left(1+qm\right)/2}}{{\left(2\pi \right)}^{q/2}}G_{\begin{array}{cc} q & 2 \end{array}}^{\begin{array}{cc} 2 & q \end{array}}\left[\frac{B^{2}q^{q}}{4}|\begin{array}{c} \Delta (q,\frac{-qm}{2})\\ \Delta (2,0) \end{array}\right]$$

Substituting *A*_5_ into (20) leads to (9) and completing the proof. □

**Proposition** **2.** *For $\bar{\gamma }\in {\mathbb{R}}^{+}$ and $\alpha ,\mu ,N\in {\mathbb{Z}}^{+}$, the following closed-form expression for the minimum sample size to achieve the desired detection performance (given ${\bar{P}}_{d}$ and $P_{f}$) over α-μ fading channels is valid*

(23) $$N=p_{PN}^{-1}\left(\frac{\mu^{2-\mu }{\bar{P}}_{d}-P_{f}}{c_{\alpha ,\mu ,\bar{\gamma }}};\frac{F_{\chi_{2N}^{2}}^{-1}(1-P_{f})}{2}\right)$$

*where $x=p_{PN}^{-1}\left(y;\beta \right)$ is the inverse of the probability mass function (pmf) of a Poisson distribution with a parameter β:$y=p_{PN}\left(x=k;\beta \right)$, (k=0,1,2,…) and β>0.*

**Proof.**  By applying Equation (5), Equation (9) can be re-written as

(24) $${\bar{P}}_{d}=\left(\underset{A_{6}}{\underset{︸}{e^{\frac{-F_{\chi_{2N}^{2}}^{-1}\left(1-P_{f}\right)}{2}}\frac{{\left[F_{\chi_{2N}^{2}}^{-1}\left(1-P_{f}\right)/2\right]}^{N}}{N!}}}c_{\alpha ,\mu ,\bar{\gamma }}+P_{f}\right)\mu^{\mu -2}$$

Therefore, Equation (24) is the closed-form expression for ROC of our proposed method. Obviously, *A*_6_ is the pmf of a Poisson distribution, namely

(25) $$A_{6}=p_{PN}\left(N;F_{\chi_{2N}^{2}}^{-1}(1-P_{f})/2\right)$$

where $p_{PN}\left(x=k;\beta \right)=\frac{e^{-\beta }\beta^{k}}{k!}$ is the pmf of a Poisson distribution with a parameter β>0. Replacing *A*_6_ in (24) by (25) yields (23) and concludes the proof. □

## 4. Simulations and Results Analysis

In this section, we verify the accuracy of our proposed expression for ${\bar{P}}_{d}$ over *α*-*μ* fading channels under a small sample size through Monte-Carlo simulation. To numerically evaluate the infinite series involved in the exact ED methods in [3,4], we must truncate the series in each expression to the same finite number of 10.

**Figure 1:**
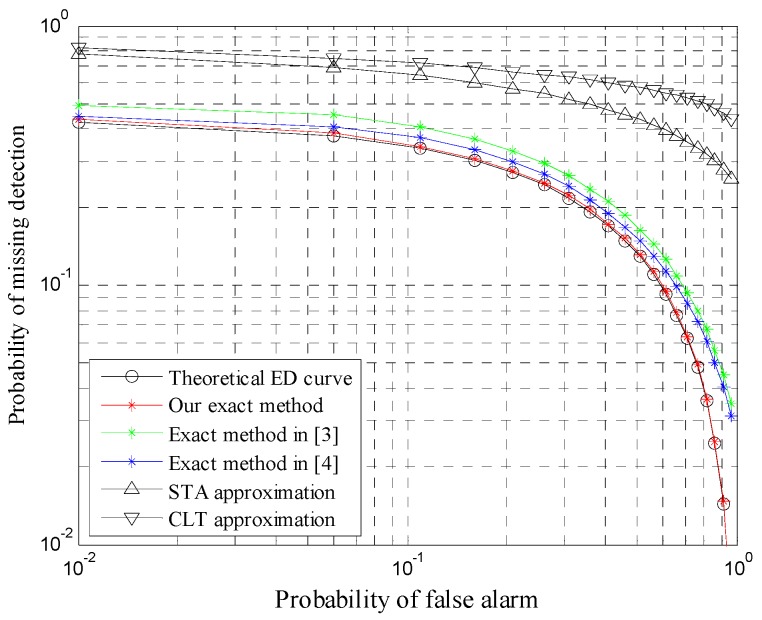
CROC curves for $\bar{\gamma }=0\;\mathrm{dB}$, α=2, μ=1.

Complementary ROC (CROC) curves of five ED schemes for *N* = 8 are illustrated in Figure 1. Obviously, the detection performance of our exact method outperforms CLT and STA approximations, especially for the low false alarm probability regime in practical scenarios. Figure 1 also demonstrates that our method is closer to the theoretical ED value than the other two exact methods. The reason lies in the fact that the two exact ED schemes need to require truncation in practice due to the infinite series, which results in some truncation errors. Therefore, our new ED scheme has the best detection performance in practical scenarios.

**Figure 2:**
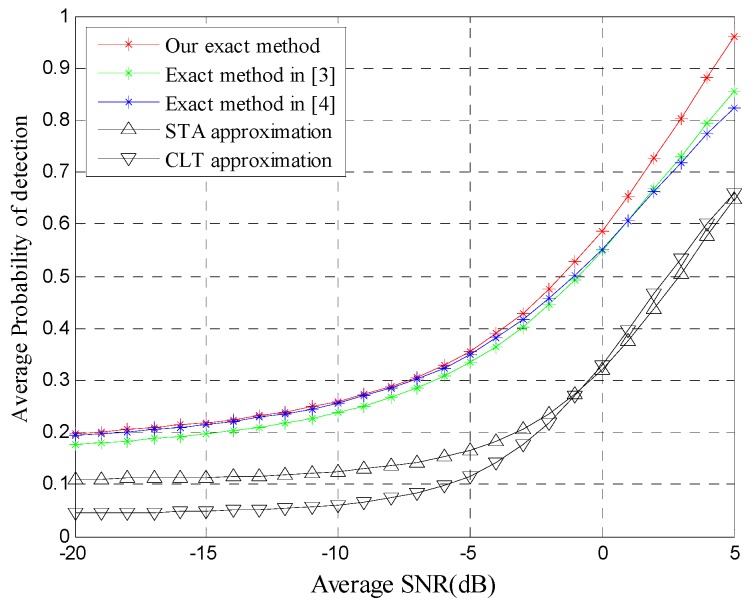
Average detection probability vs. signal-to-noise ratio (SNR) for $P_{f}=0.01$, α=2, μ=2.

Figure 2 illustrates the behavior of the average detection probability versus the average SNR for *N* = 12. Obviously, the detection performance of our exact method outperforms the other two approximations since CLT and STA are not accurate, especially for a few of the samples. To some extent, the two exact methods are actually approximations due to truncation of the infinite series. Therefore, the two exact methods are worse than our method in terms of detection performance.

**Figure 3:**
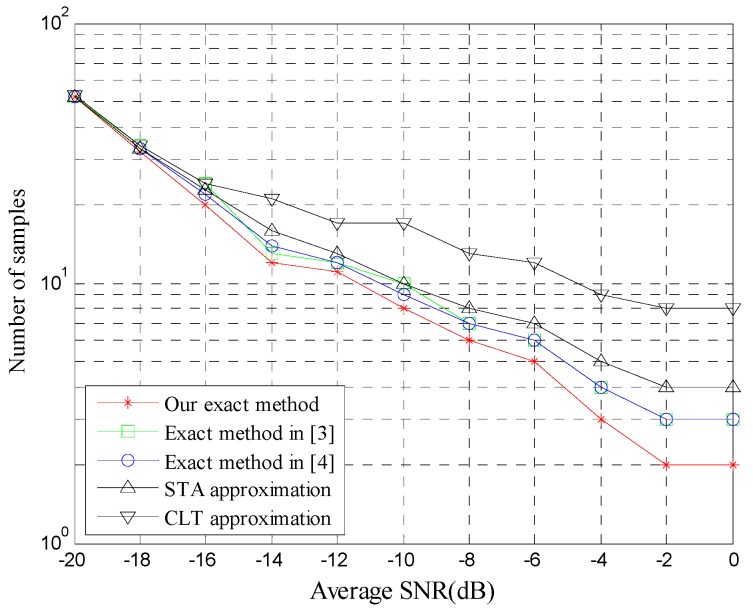
Sample size (*N*) vs. SNR for α=2, μ=2.

Figure 3 illustrates the number of samples (*N*) required to achieve $P_{f}=0.01$ and $P_{d}=0.98$, as a function of SNR. STA matches well the exact result compared with CLT. CLT is accurate only when *N* is high. In addition, compared with our method the two exact methods have small errors since the infinite series in the two methods needs to be truncated in practice. Therefore, we can conclude that with a small sample size our novel exact method is not only more accurate than the approximations, but more accurate and more practical than the existing exact methods in practical scenarios.

## 5. Conclusions

In this study, a novel exact and tractable closed-form expression for the average detection probability of ED over α-μ fading channels was derived, and then an exact and simple closed-form solution for the minimum sample size achieving the desirable detection performance was also obtained.
